# Evolution of diversity and dominance of companies in online activity

**DOI:** 10.1371/journal.pone.0249993

**Published:** 2021-04-28

**Authors:** Paul X. McCarthy, Xian Gong, Sina Eghbal, Daniel S. Falster, Marian-Andrei Rizoiu

**Affiliations:** 1 School of Computer Science and Engineering, University of New South Wales, Sydney, Australia; 2 College of Engineering and Computer Science, The Australian National University, Canberra, Australia; 3 Evolution & Ecology Research Centre, School of Biological, Earth and Environmental Sciences, University of New South Wales, Sydney, Australia; 4 UTS Data Science Institute, University of Technology Sydney, Sydney, Australia; Ca’ Foscari University of Venice, ITALY

## Abstract

Ever since the web began, the number of websites has been growing exponentially. These websites cover an ever-increasing range of online services that fill a variety of social and economic functions across a growing range of industries. Yet the networked nature of the web, combined with the economics of preferential attachment, increasing returns and global trade, suggest that over the long run a small number of competitive giants are likely to dominate each functional market segment, such as search, retail and social media. Here we perform a large scale longitudinal study to quantify the distribution of attention given in the online environment to competing organisations. In two large online social media datasets, containing more than 10 billion posts and spanning more than a decade, we tally the volume of external links posted towards the organisations’ main domain name as a proxy for the online attention they receive. We also use the Common Crawl dataset—which contains the linkage patterns between more than a billion different websites—to study the patterns of link concentration over the past three years across the entire web. Lastly, we showcase the linking between economic, financial and market data by exploring the relationships between online attention on social media and the growth in enterprise value in the electric carmaker Tesla. Our analysis shows that despite the fact that we observe consistent growth in all the macro indicators—the total amount of online attention, in the number of organisations with an online presence, and in the functions they perform—we also observe that a smaller number of organisations account for an ever-increasing proportion of total user attention, usually with one large player dominating each function. These results highlight how evolution of the online economy involves innovation, diversity, and then competitive dominance.

## Introduction

### A brave, new online world

Although now over two decades old, the web remains a relatively young platform for economic and social interactions, with new functionalities and possibilities continuously arising. Early views of the web saw the online economy as an open platform that would encourage a flourishing of diverse interests [1, 2]. However, as activity on the web has grown, attention has focused more intently on how different organisations compete for the limited attention and resources of web users [3, 4]. Several theories suggest that particular features of the web—such as positive network effects, modest switching costs and dissolving of geo-political boundaries for competition—naturally favour the emergence of online giants [5–7]. While the very existence of large internet platform companies provides anecdotal support for the competitive dominance in the digital economy, the lack of consistent, long-term data makes it difficult to see how a small number of companies have emerged as globally dominant in their respective functional domains (such as Amazon in retail, Google in search, and Facebook in social media).

The online and offline economies are intimately linked, and the economic forces that have helped fuel the growth of online giants have broader effects across the whole offline economy. For example, the fact that industries become more digitised and more connected makes them more profitable, but it also stifles competition and shields the leading firms from new rivals. This may be partially explained by these companies monopolising the attention of online users—who map back to real offline consumers—which in turn has been shown to result in an increasing dominance of a small number of firms, who are more profitable and face less new entrants [8]. Online attention has been previously measured through metrics such as counts of online searches [9], video views [10, 11], retweets [12, 13] or webpage links [8], and has been shown to predict patterns of offline behaviour [14] accurately. For example, the volume of specific web search terms has been shown to correlate to the real world social and economic phenomena such as unemployment [15], housing price trends [16] and the relative market shares of sales of companies in emerging markets such as electric car brands [14]. Search traffic on specific terms [17], visit counts [18] and edits made [19] to specific Wikipedia articles and terms used in Twitter posts have also been shown to correlate to population dynamics (for example, the spread of influenza [20]). As always, caution is needed in interpreting these results, as it is impossible to demonstrate causative links from observational data of large complex systems. Yet few would deny that a company’s online presence could be causally linked to its long-term success.

New commercial and social uses for the web—dubbed here as *functions*—are continually being explored and developed. Often new technologies enable such new functions to emerge. For example, the advent of secure data communication on the web set the stage for e-commerce and for companies like Amazon to emerge; the roll out of broadband-enabled video enabled applications such as Youtube and Netflix to grow; further, the democratisation of mobile terminals (i.e. smartphones) gave rise to location-based applications such as Uber and Airbnb. Peer-to-peer accommodation sharing, ride sharing and ephemeral social media are three of the recent innovative functions. As new functions emerge online and a field of new competitors assemble, the relative growth in companies’ revenue in a function can indicate which company will come to dominate (and which can be under some circumstances modelled and predicted [21, 22]). Revenue in turn is a function of growth and highly dependent on the retention of customers [23] and, particularly, their attention [24]. As a result, the early distribution of online attention within a function can be seen as a leading indicator of the company that will dominate the function.

Between 2006 and 2017, the web has grown significantly in terms of its number of users, average usage time by the users and the number of organisations active online. For example, the number of internet users worldwide has grown from 1.2 Billion 2006 to 3.3 Billion in 2016 [25]. Similarly, the amount of time spent online grew steadily over the years, from 2.7 hours per day in 2008 to 5.7 hours to per day 2016 in the US [26]; Finally, the number of organisations, brands and services represented online has grown dramatically. The total number of domain name registrations grew from 79 million in 2006 [27] to 329 million in 2016 [28]. Network theory shows that the web grows following the law of increasing returns [29] where new links are added where others already exist (also known as power-law or preferential attachment, also shown in our data, see Materials and methods). This phenomenon leads to a cumulative advantage for a small number of organisations (and their online services) that enjoy most of the attention, while the others attract very little [30]. Network topography has also been shown to govern user attention and activity, which also follow a power-law distribution [31], as does the number of users across websites relative to their rankings by total visits, and the total attention given to each of these websites [32].

We leverage the above-mentioned network theory insights, combined with a novel data source (see Materials and methods), to study the rise and fall of attention given to businesses online. To quantify the dynamics of activity online, we present a longitudinal study of user attention on two popular social media sites—Reddit and Twitter. Our analysis spans over 10 years of Reddit history and more than 6 years of Twitter history. Both Reddit and Twitter allow users to share ideas and links on topics of interest, with 310 million and 328 million global users respectively [33]. As such, these public platforms provide a chronicle of internet users’ attention and interests over time. Note that we do not follow individual users, we measure the aggregated attention patterns *towards* companies. We use the volumes of outbound links posted to a companies’ website as a proxy for user attention towards the organisation and its products.

Employing weblinks has several advantages. First, they are structured around domain names. Usually, each domain corresponds to a single company—in this work, we use *domain* to interchangeably denote a web domain or a company with an online presence. Second, weblinks are easily identifiable from the surrounding natural language text and easily tallied. Third, links are central to the web’s architecture, and link counts are significant indicators of the website quality and authority. They are also at the heart of the ranking in search indexes, including Google’s PageRank algorithm [34]. In fact, our present work shows that the PageRank of domains and the online attention quantified from social data are distributed very similarly (see Results and discussion and Fig 2). In an increasingly global digital economy, online attention is a new form of currency. One key measure is online links, which are shown to drive attention which in turn drives online dominance which, we postulate, is linked to market dominance.

There are several reasons why unconventional data, such as social media links, are a beneficial and useful source of information for researchers interested in business dynamics. In addition to being publicly available and timely (and massive in scale) these data have at least three advantages over traditional financial market data. First, for new and emerging categories, traditional financial measures are often unavailable or not meaningful. Second, sometimes the direct competitors are not known. For examples, the competitors are often private companies part of broader business conglomerates with multiple revenue streams; therefore, individual functional business unit financial data is not available. Third, in some cases, especially in emerging and new industries such as electric vehicles, trends in social media data may precede, anticipate or even predict trends in businesses, financial markets and the broader economy.

### Competitive diversity and its role in economics

In Economics, a variety of independent firms competing with each other in each market and segment of the industry is vital for the economy’s health. Diversity is at the heart of all effective competition and, in turn, has been shown to lead to higher rates of productivity growth [35]. The number, structure and variety of competing firms in a sector in Economics can be seen as analogous to the diversity of species in Ecology, in a niche. If there are too few competitors or a small number of players become too dominant within any economical sector, there emerges the potential for artificially high prices (monopoly rent) and constraints to supply. Even more importantly, in the long-term, this gives rise to constraints on innovation. Nobel-winning economist Ken Arrow [36] postulated that, once markets are dominated by an established firm or group of firms, this establishment has less incentive to innovate. This is because they would have an added cost to innovation that an innovating competitor would not—the opportunity to continue to earn monopolistic profits without innovating.

We explore the large-scale and long-term trends in industry structure and economic variety through the lens of online attention in several key dimensions, including:

- scale—how many different organisations are active online;
- originality—how many distinctive sources of information and services are there online;
- diversity—how attention is divided and distributed between firms online.

We also examine online innovation through the birth of new online business functions, such as ride-sharing, online video, and ephemeral peer-to-peer messaging. We propose that competition among organisations online follows a three-phase dynamic [5]:

1. Infancy—after the emergence of a new function, we observe a great burst of diverse businesses that appear and start to serve the function;
2. Development—this phase begins once the number of competitors within the function peaks and begins to dwindle, as competitors start to lose market share to one another;
3. Maturity—during which we see a reduced diversity in the function, as the majority of users converge around a single dominant organisation.

## Materials and methods

In this work, we longitudinally integrate and analyse three types of data sources: 1) proxies for online attention towards companies, 2) the list of competitors for online companies and 3) the economic performance of companies. We measure **the attention towards companies online** by examining links posted over a decade from two major online platforms: Reddit (www.reddit.com) and Twitter (www.twitter.com). We use social media data as a proxy for online human attention towards companies. We also use Common Crawl (https://commoncrawl.org/), which records the PageRank [34] over time, and which can be seen as a proxy for the attention received from internet websites. We combine and index these datasets with two other data sources which record **competitor data**: Crunchbase (https://www.crunchbase.com/) and Rivalfox (which closed in 2017, but for which the competitor maps are still available via the Internet Archive—e.g., for Airbnb competitors from Rivalfox see https://web.archive.org/web/20150327025122/https://rivalfox.com/airbnb-competitors). Finally, we measure the companies’ **economic performance** by building the *enterprise value* using historical data from Financial Times (https://www.ft.com/) and Yahoo Finance (https://finance.yahoo.com).

### Data collection

Here we discuss the collection of online attention data (social media and webpage linking) and economic performance data. All the constructed longitudinal datasets and all the code required to reproduce the research and the figures in this paper are publicly available online at https://github.com/behavioral-ds/online-diversity.

#### Social media data

**Fig 1:**
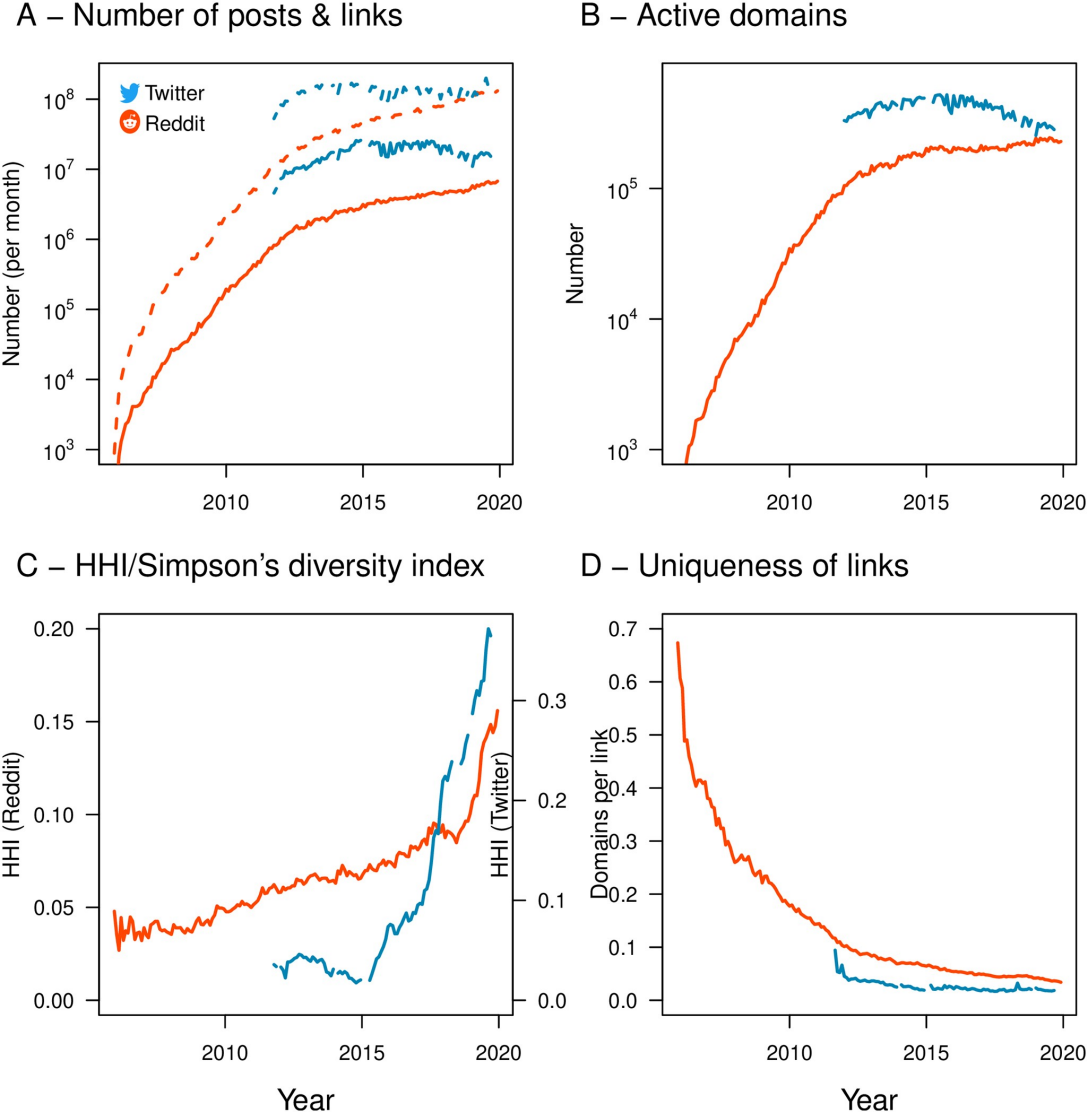
Dynamics of activity on online platforms, as indicated via posts in social media platforms. **(A)** Growth in the number of posts (dotted) and links within posts (solid) in both Reddit and Twitter over time. **(B)** The number of distinct active domains appearing within social-media links to has also grown. **(C-D)** An increase the HHI index (C) and a decrease in link originality (D) for domains within links indicates that, despite the growth in total activity (A-B) diversity in online activity is in long term decline (see Sec. Materials and methods).

From Reddit, we used the publicly available dumps of Reddit comments (see http://files.pushshift.io/reddit/comments/), which are claimed to capture all Reddit activity from December 2005 until December 2019. Each dump file contains all the comments posted on Reddit during one month, in JSON format. The available fields include the posting date, the author and the text of the comment. We compiled and analysed more than 10 years worth of data, comprising more than 6 billion Reddit user comments. For Twitter, we used data from a long-running crawler leveraging the Twitter Sampled stream (retired as of Oct 2020, see https://developer.twitter.com/en/docs/labs/sampled-stream/overview), which returns in real-time a sample of 1% of all public tweets. The crawler ran continuously from September 2011 until the end of 2019, and the datasets contains several gap periods (seen in Fig 1 and the S1 File), when network errors took the crawler down. We compiled and analysed more than 11.8 billion user posts (known as tweets), published from September 2011 to September 2019 (see Table 1 for datasets stats).

**Table 1:** Summary of the dimensionality of our datasets: The number of posts, links contained in posts, and unique domains linked in each of the two datasets. For reference only, we also show the size of the dataset in occupied disk space (both datasets are compressed with the BZIP2 utility). The combined number of domains (indicated by *) is the number of unique domains in our datasets.

Dataset	# posts	# links	# domains	Dataset size (TB)
Reddit	6,095,691,657	353,915,452	4,377,343	0.34
Twitter	11,813,340,769	1,553,959,383	10,152,547	5.25
Common Crawl	-	19,989,755,161	90,983,688	0.03
Combined	17,909,032,426	21,897,629,996	95,709,245*	5.62

#### Common Crawl web linking data

We compile the linkage record over time of online websites towards domains using Common Crawl (https://commoncrawl.org/). Common Crawl is the largest open index of the web and which has been shown to represent over 80 per cent of the world’s most popular public web sites when compared with data from Amazon (Alexa) and other sources [37]. The raw data record a large array of linkage features for each quarter between May 2017 and May 2020. The 69 million most linked domains, Common Crawl computes the PageRank for each domain, which we use as a proxy for the attention received from internet websites: the higher the PageRank, the more central a domain is and therefore the more attention (weblinks) it receives.

#### Economic performance data

For one case study, we aim to examine the relationship between the enterprise value of electric vehicle manufacturer Tesla and the online attention that it receives. We collected time-series data relating to publicly listed electric car maker Tesla, Inc (NASDAQ:TSLA) between December 2015 and September 2019. The enterprise value for Tesla was sourced by using the current market capitalisation for Tesla from Financial Times (https://www.ft.com/) in USD and we used the adjusted historical time-series share price data (Yahoo Finance, https://finance.yahoo.com) to determine historic enterprise value of Tesla, adjusted for both dividends and splits.

### Measuring the spread of online attention

Here, we further detail the measurements we perform to quantify the dynamics of online attention allocation. First, we look at the distribution of online links in social media and model their best fit. Next, we investigate its concentration over time using several indicators. Finally, we propose a simple measure of *link originality* to measure online diversity, and we examine its evolution over time.

For both Reddit and Twitter, we tally the number of posts and outbound links within user posts during observation periods of one month. Outbound links point to websites and online services outside the host platform. Next, we classify each link based on its major domain—for example, a link such as https://www.youtube.com/watch?v=dLRjiiAawGg refers to the domain www.youtube.com, which belongs to the online video hosting platform Youtube. We consider a domain as being active if it is linked to at least once within an observation period.

#### Measures of attention diversity

To understand and quantify patterns and trends in the diversity of online businesses, we use a measure that is common to both Ecology and Economics—known as the Simpson Index [38] in Ecology, and the Herfindahl-Hirschman Index (or HHI) [39] in Economics. We use HHI to measure the diversity of online domains based on the number of links that point to them. Formally

$$\begin{array}{c} HHI=\sum_{i=1}^{N}S_{i}^{2} \end{array}$$

where *S*_*i*_ is the percentage of all the links within a platform relating to domain *i*, and *N* is the number of distinct domains at each period in time. HHI values range from zero to one. The higher the HHI value the less evenly distributed the links are. HHI values that are very low and close to 0 represent when links are evenly distributed between domains (perfect competition is achieved when all firms have equal shares). Conversely, high HHI values represent a very uneven distribution of links (for complete monopoly, the HHI is 1 when one firm attracts all the links). Due to the size of our datasets, HHI was computed on a cluster computing environment using the R programming language [40], using the package DescTools.

We also propose a new measure to quantify the bias in the distribution of attention—dubbed *link originality*—defined as the average number of domains per link. Link originality takes values between ≈0 (absolute monopoly, all links stem from the same domain) and 1 (complete diversity, each link has a distinct and original domain).

The logic behind link originality is as follows. A known measure for the skewness of a distribution is the difference between the mean and the median value. Even though some domains feature millions of links per month, half of all domains in our datasets are linked at most once each month—i.e., the median number of links per domains is one (1) for each analysed time frame. Therefore, we can measure the increasing skewness of the attention distribution by tracing the average number of links per domain comparatively to the fixed median of one. Link originality is defined as the ratio of number of domains to the number of links, and it is the inverse of the mean attention received by domains. Link originality is a simple, intuitive measure for online diversity: when originality decreases, mean domain attention increases, the difference between mean and median attention increases, indicating that the distribution is increasingly skewed.

#### Measures of attention concentration using PageRank and CommonCrawl

We measure the spread of the “attention of webpages” using the CommonCrawl dataset, which records the PageRank for each domain. PageRank quantifies the inbound links to a page to determine how important a website is, assuming that more important websites are likely to receive more links from other websites. The PageRank for all domains in each period adds to one—an intrinsic property of the PageRank. Summing the PageRanks of the *top n* domains in each period forms an effective measure for the relative market share of total inbound links to each these ‘important domains’ over time. Even though the composition of *top n* will change over time, their total PageRank in each period can reveal the broader trends of link concentration.

### Linking social media attention and enterprise value

To explore, whether there is a link between attention on online social media platforms towards particular companies and their enterprise value, we use the time-series financial market data for the electric vehicle manufacturer Tesla, and the count of link counts posted on Reddit and Twitter towards its domain. We chose the Tesla case study given that previous research has shown that a growth in links in social media is predictive for the growth in sales and market share of electric car brands [14].

We use statistical analysis to explore whether a growth in links on social media is predictive of a growth in enterprise value for Tesla. We use three time-series data relating to publicly listed electric car maker Tesla, Inc (NASDAQ:TSLA) between December 2015 and September 2019. The first series is the Enterprise Value (EV) of Tesla. The second and third series are the counts of outbound links to tesla.com on Reddit and Twitter, respectively. We perform the analysis in two steps. **In the first step**, we perform stationarity tests to see whether the mean, variance, and autocorrelation for each time series are stable over time. We use the augmented Dickey-Fuller test (ADF), and we obtain that only the Twitter series appears stationary (p-value <0.05). We transform all series by *differencing*—i.e., compute the differences between consecutive observations—which renders them stationary. Next, we perform co-integration tests to estimate the long-term equilibrium of two series in order to rule out the possibility of spurious correlation. We obtain that none of the two pairs (Reddit and EV, Twitter and EV) are co-integrated (see the S1 File for more details).

**In the second step**, we examine whether links in these social media data sets (Reddit and Twitter) can be used to forecast the changes of enterprise value using Granger-causality. We vary the lag parameter in the Granger-causality test between 1 and 12 periods (corresponding to 1 to 12 months), and we obtain that for a number of lags larger than two (for Reddit) and four (for Twitter), the granger-causality test is significant (p-value <0.05). Finally, we use vector auto-regression (VAR) to determine the optimum number of lags by selecting the value which minimises the Akaike information criterion (AIC). We obtain that the optimal lag is two for Reddit and four for Twitter. Further details of each of these steps and their results are included in the S1 File.

### Categorising new functions and innovation in the online economy

The widespread adoption of key digital platform technologies such as security, mobility and broadband themselves enable waves of new business opportunities to emerge. Platform technologies create the conditions to offer services that could not be offered previously for example when security was added to the web it moved from being an information medium to becoming a transaction medium and, in the process, enabled a plethora of new commercial services such as online retail, online payments and online banking to emerge.

By following the parallel with the field of Ecology, new *niches* (dubbed here *functions*) emerge and new companies quickly move in to seize the opportunity. We operationalise the concept of online functions using the Crunchbase functional categories [41]. Crunchbase (www.crunchbase.com) is an index of companies, for whom a series of indicators are recorded, such as its location, number of employees, the funding rounds and the amount of money raised. Crunchbase classifies companies using one or more labels from a taxonomy that records 744 categories, which are intended to correspond to specific market segments. We study twelve such categories (or functions): Social network, Search, General retail, Filesharing, Music streaming, Movies & TV, Ride sharing, Accommodation, Action cameras, Ephemeral messaging and Dating Apps for mobile. We find that the Crunchbase categories are very broad, encompassing companies whose main business does not relate to the function (e.g. ‘Mattermost’ is also listed as ‘File Sharing’, when its main function obviously is ‘Messaging’).

We perform a second pass of selection. For each function, we study the top companies (based on the total volume of links in Reddit) and we manually select a ‘champion’ which aligns most closely with the function (such as Uber, Spotify, AirBnb or Dropbox). Next, we identify their top three ‘rivals’ as of January 2017—using Rivalfox (now closed). This results in a curated list containing twelve functions, and the four main rivals in each function. For example, the function ‘Ride sharing’ contains Uber, Lyft, Hailo and Sidecar (see the S1 File for the complete list of functions and rivals.). Finally, we record the date of the first link towards a company in that function—i.e. the date that the function emerged—and the total number of links towards companies in that function—the total attention towards the function.

We also study the increasing online competition for different temporal cohorts based on their survival rate. We group online companies based on the year when they are first linked in Reddit, and we build 11 temporal cohorts (one for each of the years 2006 to 2016). We follow the companies in each temporal cohort as they age, and we keep track of how many of them are still active—i.e., have at least one link during a one-month observation period. Note that the survival rate can increase, as some domains can remain dormant and not be linked during one or several months. In an equal opportunity environment, the survival rate at equal ages should be similar. However, as activity on the web grows, we expect competition to become more intense as the number of key functions having reached the maturity phase grows. As a result, we expect the survival of new domains in the webspace to be lower.

## Results and discussion

We first analyse the dynamics of online attention and the observed reduction of online diversity. Next, we study the growth of online functions and, finally, the dynamics of temporal cohorts. Our analysis of online attention towards companies on two large social media websites reveals a number of consistent trends.

Since online attention is a proxy for global users attention to online services and platforms such as Youtube, Etsy and Amazon, but also to offline businesses and brands such as Disney, Tiffany & Co and Walmart, our results could potentially shed light upon the broader economic trends in the era of the web.

In one select case study, we show that the volume of online attention to the company Tesla is predictive of its enterprise value four to twelve months in advance.

### Dynamics of online attention in social media

During the last decade, the activity on Twitter and Reddit has increased exponentially (seen in Fig 1A, notice the log y-axis). At the same time, the total number of active domains on Reddit—i.e. domains that have been linked at least once during a one-month period—has increased at least two orders of magnitude from 1000 in 2006 to over 10,000 in 2020. The number of distinct and active domains linked to on Twitter is much higher and almost doubling from 246,000 in 2011 to 447,000 links per month (Fig 1B). Despite these results being consistent with the growth patterns of the web as a whole, we observe at the same time a long term decline in the diversity of services that makes up the online activity. We measure in Fig 1C the Herfindahl-Hirschman Index (HHI) and in Fig 1D the link uniqueness (see Sec. Materials and methods for more details). Both figures convey the same message: in both Reddit and Twitter we observe an increasing attention concentration over time indicating that an ever increasing proportion of users attention that is focused on a smaller and smaller percentage of popular domains.

#### Measuring the concentration of online attention

**Fig 2:**
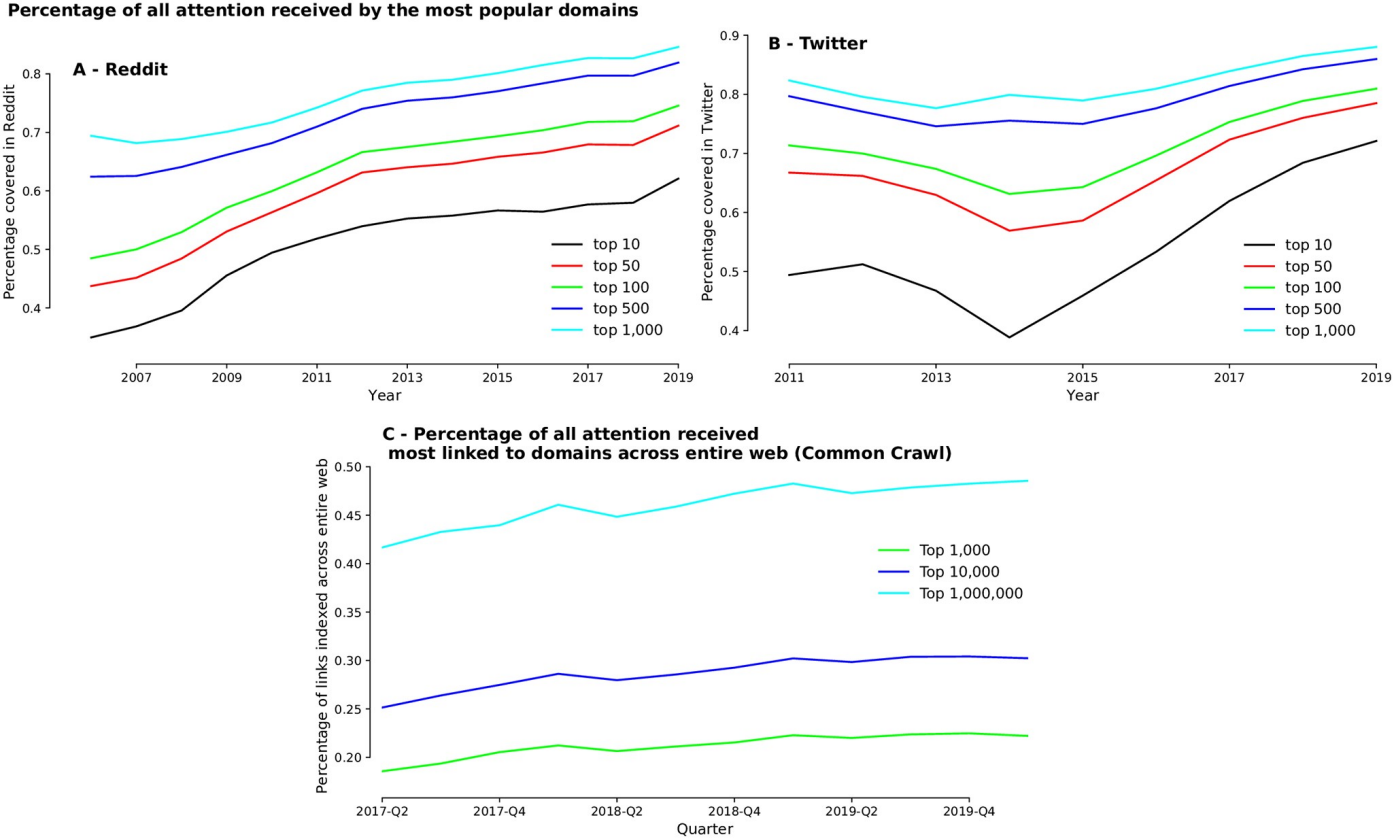
Top websites attract growing percentages of overall links across the entire web. **(A)(B)** Percentage of all links associated with the most popular 10, 50, 100, 500 and 1,000 domains in Reddit (A) and Twitter (B). The top 10 most popular domains in Reddit received around 35% of all links in 2006, which grew to 60% in 2019. In Twitter, the top 10 domains grep from 50% (2011) to 70% (2019). **(C)** The sum of PageRank values for the top 1,000, 10,000 and 1 Million domains respectively within the Common Crawl corpus.

We can detect the temporal increase of dominance by tracking the change over time in the percentage of attention captured by the most popular domains. We plot in Fig 2 the percentage of total attention received by the *top n* domains, with *n* between 10 and 1,000 for Reddit and Twitter. For Reddit, Fig 2A shows that the top 10 most popular domains received around 35% of all attention in 2006, which grew to about 60% in 2019. The percentage of attention to the top 1,000 domains (out of the total of more than 3 million on Reddit) is above 80%. For Twitter (Fig 2B), the concentration is even more pronounced, with the top 10 domains commanding about 50% of all attention in 2011 and more than 70% in 2019, and the top 1,000 reaching between 80% and 90% of all attention. Overall, these results indicate that online media attention is very concentrated on a handful of domains, and getting increasingly concentrated over time. Noticeably, Twitter saw a period of reduction of concentration around 2014 which was reversed towards the end of the dataset timeline.

In search for independent evidence of this concentration, we study the concentration of online attention of webpages using Common Crawl by investigating the changes in the market share of links to the top domains over time. Fig 2C shows the total market share of links—defined as the sum of their PageRank—to the *top n* most popular domaine (top 1,000, top 10,000 and top 1 million). Similar to Reddit and Twitter, we can see that each of these grows consistently over time, clearly illustrating a growing concentration of links across the entire web among the most centrally-linked and dominant websites. We also observe that top 1 million domains has now grown to represent almost half of the market for links in 2020. Even though the spread of total market share between top 10,000 and 1 Million is relatively large (around 17.4%), the spread between top 1,000 and 10,000 is only about 7.5%, also indicating that the top 1,000 domains occupy almost the half of the attention of the top 1 Million.

**Fig 3:**
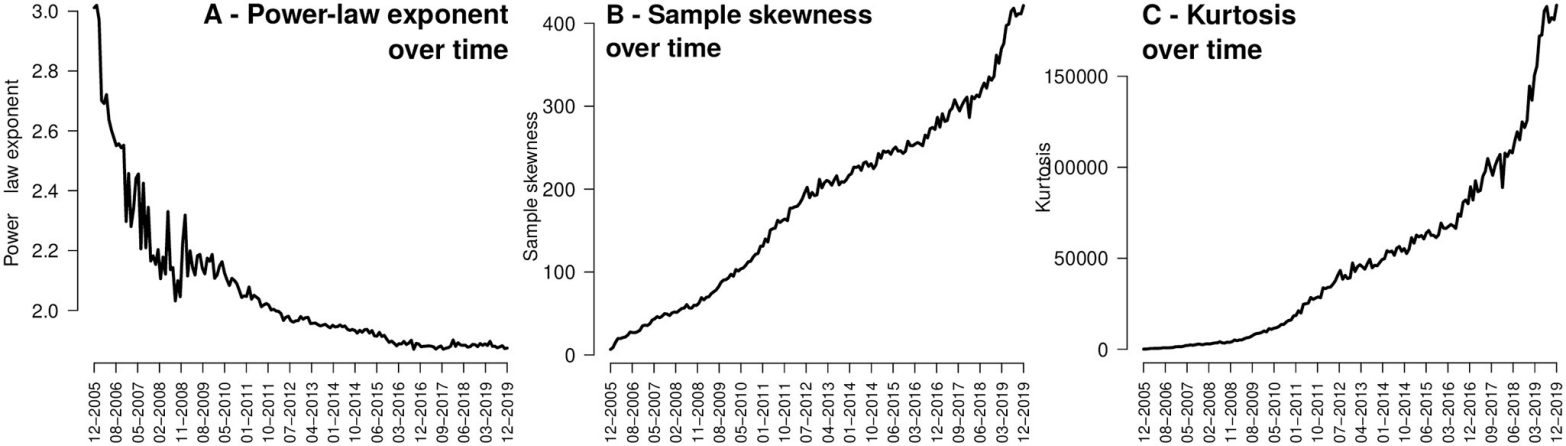
The distribution of user attention (i.e. number of links per active domain) in Reddit, over domains, is getting increasingly more skewed over time towards the popular domains: *The rich are getting richer*. **(A)** we fit a power-law distribution and we plot the exponent of the power-law distribution over time; it shows an decreasing trend over time (i.e. higher inequality). **(B)** and **(C)** we compute the sample skewness and kurtosis respectively; both show a upwards trend over time.

Next, we measure the concentration of attention from the point of view of the skewness of the attention distribution. We compute the attention towards domains for each one month time interval, and we measure the skewness and the kurtosis for each of these distribution. These are both widely used measures of distribution. A positive skewness value indicates that the tail on the right side is longer or fatter than on the left side and high kurtosis values are the result of infrequent extreme deviations (or outliers), as opposed to frequent modestly sized deviations. Large positive values for both measures indicate a highly skewed distribution (long-tail), the larger the more skewed. Fig 3B and 3C illustrate the skewness and the kurtosis for Reddit. Both measures show increasingly higher values with time, indicating that attention is getting more and more dominated by several (few) domains.

#### User attention to online domains is long-tail distributed

**Fig 4:**
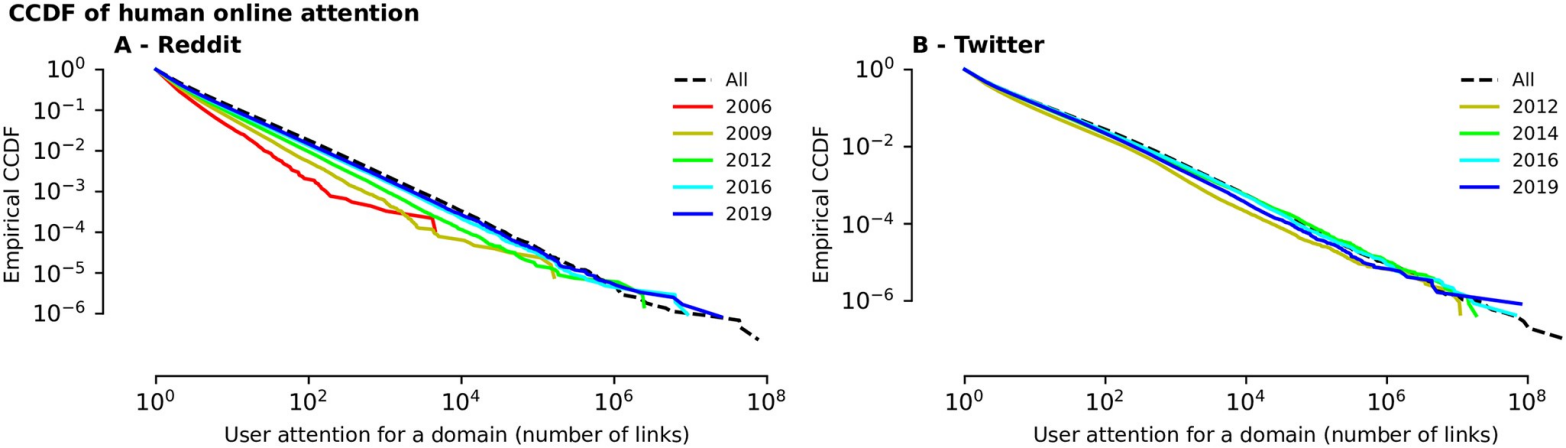
Cumulative attention in social media (Reddit and Twitter) follow a rich-get-richer distribution (i.e. long-tail), which get even more skewed over time. The log-log plot of the empirical Complementary Cumulative Distribution Function (CCDF) of the number of links associated with domains in both Reddit **(A)** and Twitter **(B)** appears almost linear—a sign of a long-tail distribution. Each solid line shows the distribution of links posted during a particular year, while the dashed line shows the distribution for the entire dataset.

The above observations indicate that online attention is long-tail distributed, with an increasingly longer tail over time. Such distributions are also known as “rich-get-richer”, because most of the total attention is captured by a small number of online domains. To test this hypothesis, we plot in Fig 4 the log-log plots of the empirical Complementary Cumulative Distribution Function (CCDF) of the number of links for domains over time, in Reddit and Twitter. Visibly, the CCDF appears linear, which is indicative of the long-tail distribution of attention. We also analyse the distribution of attention with given time periods, by counting only the links that were posted in particular years (here 2006, 2009, 2012, 2016, and 2019 for Reddit and 2012, 2014, 2016 and 2019 for Twitter). The attention pattern referred to the overall period and to any of the selected years and the distribution lines associated with later years are shifted right-upwards, since Reddit grows as a whole. Unlike Reddit, Twitter does not shift, as Twitter as a whole peaks in size in 2016, and decreases ever since (shown partially in Fig 1A, and in the zoom-in in the S1 File).

We search for a theoretical long-tail distribution that fits best the distribution of online attention. We fit the data to several theoretical long-tail distributions (including power-law, exponential and log-normal) [42], and we observe that the power-law distribution has the tightest fit measured using log-likelihood ratios over the studied periods of time (see the detailed analysis in the S1 File). Next, we set to detect the concentration of online attention via the fitting of power-law distributions for each of the monthly attention of domains. Let X be a random variable, the attention received by a domain during a given time period. The probability function *P*(*X* = *x*) decays as a power-law with parameter *α*, which controls the speed of the decay. This can be interpreted as follows: for high *α* values, the probability of observing of a domain having received more attention than *x* (the CCDF *P*(*X* ≥ *x*)) decreases faster than for lower values of *α*. Consequently, for large *α* it is less likely to have very popular domains, i.e. less dominance and more diversity. Conversely, lower *α* is indicative of more dominance and lower diversity. Fig 3A plots the evolution over time of the fitted value of *α*. Visibly, the *α* exponent is decreasing over time, which implies that the existence of massive giants is increasingly likely.

In conclusion, the above observations show that despite the fact we observe dramatic growth of the overall web and in major social platforms such as Reddit and Twitter, we also see at the same time a long term decline in the diversity of services that makes up this online activity, with increasingly fewer players controlling increasingly larger shares of the market.

### Growth of functionally diverse services

Business innovation is often a result of the emergence of new enabling platform technologies—such as geolocation, security and broadband combined with a critical mass of users with access to this technology. For example, online *General retail* has been enabled by the roll-out of secure online payments; online *Video* with more broadband access and others like *Ride sharing* with the widespread adoption of smartphones.

Many services themselves enable others to flourish too. For example before smartphones, the advent and widespread adoption of *Webmail* services and public internet cafes enabled large numbers of travellers to check email and adjust and rebook travel plans while on the move thus advancing the growth of online *Accommodation* services.

Others are an effective and orchestrated combination of a range of other technologies. *Ride sharing*, for example, has mixed geolocation, secure payment and instant messaging to create a global alternative to the taxi industry. These foundation online technologies and services form the basis to enable more and more complex new services to be offered online, and their networked nature means the widespread adoption and diffusion of new services is increasingly rapid.

In this section we analyse the dynamics of the online attention economy in waves of innovation. We show the temporal emergence of online functions and we study the dynamics of the survival rates for online companies.

While the web started as an information media, with the addition of security, mobility and broadband access it has quickly evolved as a marketplace for services. Waves of business and services innovation have followed each wave of infrastructure innovation and we identify four such waves.

**In the first wave**, simple text based information services emerged such as an online reference about the cast and crew of almost all movies ever made (IMDB 1990), a platform for online diaries (Blogger 1993) and a service for online classifieds (craigslist 1995). **In the second wave**, with addition of online cryptography and security, a raft of new commercial services emerged such as Online Retailers (Amazon 1994); Online Classified Auctions (eBay 1995) and Online Payment (PayPal 1998). **In the third wave**, the ability for large number of internet users to be connected to high-capacity broadband led to more innovation in media and communications services such as Online Telecommunications (Skype 2003), Online DIY Video (YouTube 2005), and Online Movies (Netflix Streaming 2007). Finally, **in the fourth wave**, the advent of online mobility created services to track movement online (fitbit 2007); create new marketplaces for accommodation (Airbnb 2008) and ridesharing (Uber 2009).

We postulate that the above-defined waves of technology innovation lay the foundations for new types of services or functions to emerge. These new functions compete not only with other companies on what they are selling but in the ways of delivering products and services and thus are often disruptive to existing players as they circumvent existing approaches to market. New functions also provide new avenues for emerging companies to enter the market and often a key part of the competitive advantage of startups. Whether delivered by startups or established firms who reinventing themselves via digital transformation, new technology-enabled functionally diverse services forms the basis for much of the disruptive innovation we have seen over the past two decades.

**Fig 5:**
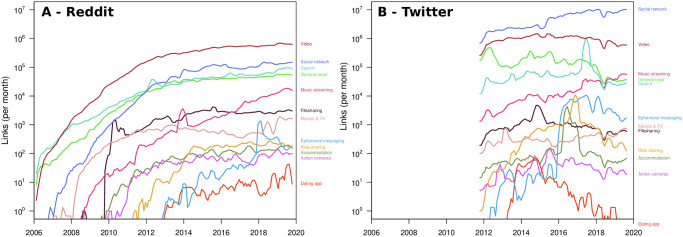
Growth of functional diversity on the Reddit (A) and Twitter (B) platforms. The lines shows the number of links to different functions as indicated by the leading organisations and their top-three rivals (see *Sec. Categorising new functions and innovation in the online economy* for details).

Our measurements provide empirical proof for this hypothesis, as they show that different functions appear at different times, and they follow similar patterns of increasing activity. Fig 5A shows that in Reddit new functions continuously emerge and the attention towards each of them grows consistently, exponentially at first (note the log scale of the y-axis) and at more moderate rates as the functions mature. Fig 5B provides comparable conclusions for Twitter, but over a shorter timeframe. Note the relative decline in links to some functions on the largely mobile user base of Twitter such as dating, accommodation and filesharing—possibly due to the widespread uptake of mobile apps in these areas.

### Survival rates for newborn online services are in decline

For a company, the success in securing online attention correlates with business success. Conversely, the lack of online attention can signal a decline in customer demand or defunct services. In our analysis, a domain’s ‘birth’ occurs with the first link to have ever been posted towards the domain, while its ‘death’ occurs with the last link that points to it—obviously there is an edge effect at the end of our dataset in 2019, which we account for by not over-interpreting the results for the last cohort of 2018.

**Fig 6:**
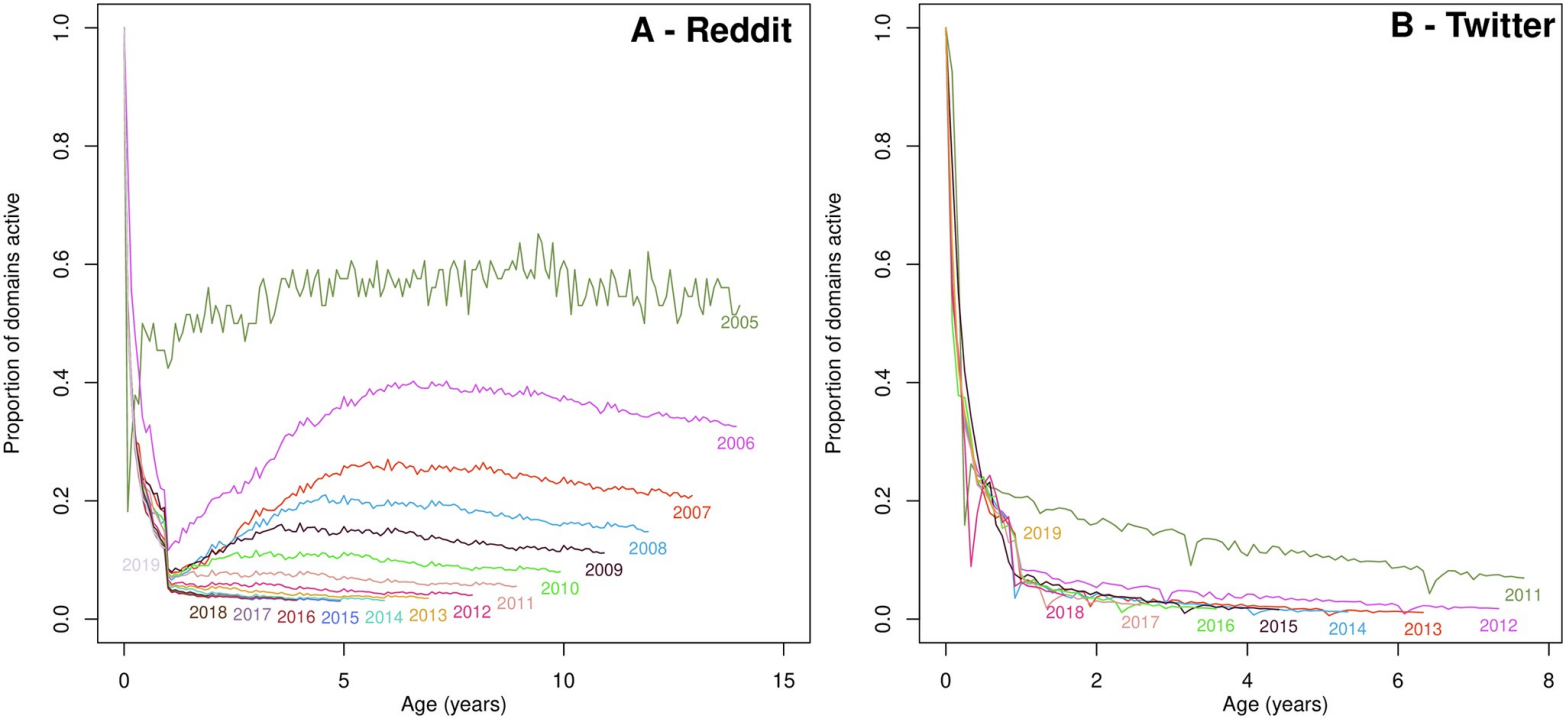
Survival rates of newborn online services are in decline in both Reddit (A) and Twitter (B). Lines show survival rates for the cohort of all new domains sorted by year of first appearance. The percentage still active 5 years after their first appearance from that birth year is in steady decline. Survival may increase after year 0.

In Fig 6, we group domains in temporal cohorts based on the year of their birth (see Sec. “Categorising new functions and innovation in the online economy”). Each line in Fig 6 corresponds to a temporal cohort, and we observe that survival rates of newborn online services are in decline. For example, the percentage of domains still active 5 years after their birth year has declined from just under 40% for the 2006 cohort to a bit over 3% for the 2015 cohort. That is to say, a smaller proportion of newborn domains survive to older ages in later cohorts, which indicates that the competitive environment for young firms is becoming more hostile over time.

Consistent with previous research that shows that growing digitisation of industries stymies new entrants [8], our analysis reveals *mortality rates* of new entrants online are on the rise. This indicates the environment for new players online is becoming increasingly difficult. In the way pine trees sterilise the ground under their branches to prevent other trees competing with them, once they are established dominant players online crowd out competitors in their functional niche.

Let’s take search as an illustrative example. Google was founded in 1998 and fought off many early rivals such as AltaVista, Yahoo and Hotbot to the crown of the world’s search engine of choice. Another serious competitor to Google, Cuil, emerged late on the scene some eight years later in 2006 but by this stage Google’s market share was clearly dominant. Despite attracting over $30 million in investment from leading Venture Capital firms and indexing more websites than Google, Cuil was unable to make the slightest dent on Googles market share—achieving only 0.2 percent of worldwide search engine users in July 2008 and the service was shut down in 2010. Consequently, Google has gone on to dominate search in the English-speaking world with over 90 percent market share in the past decade [43].

### Linking online attention to enterprise value

**Fig 7:**
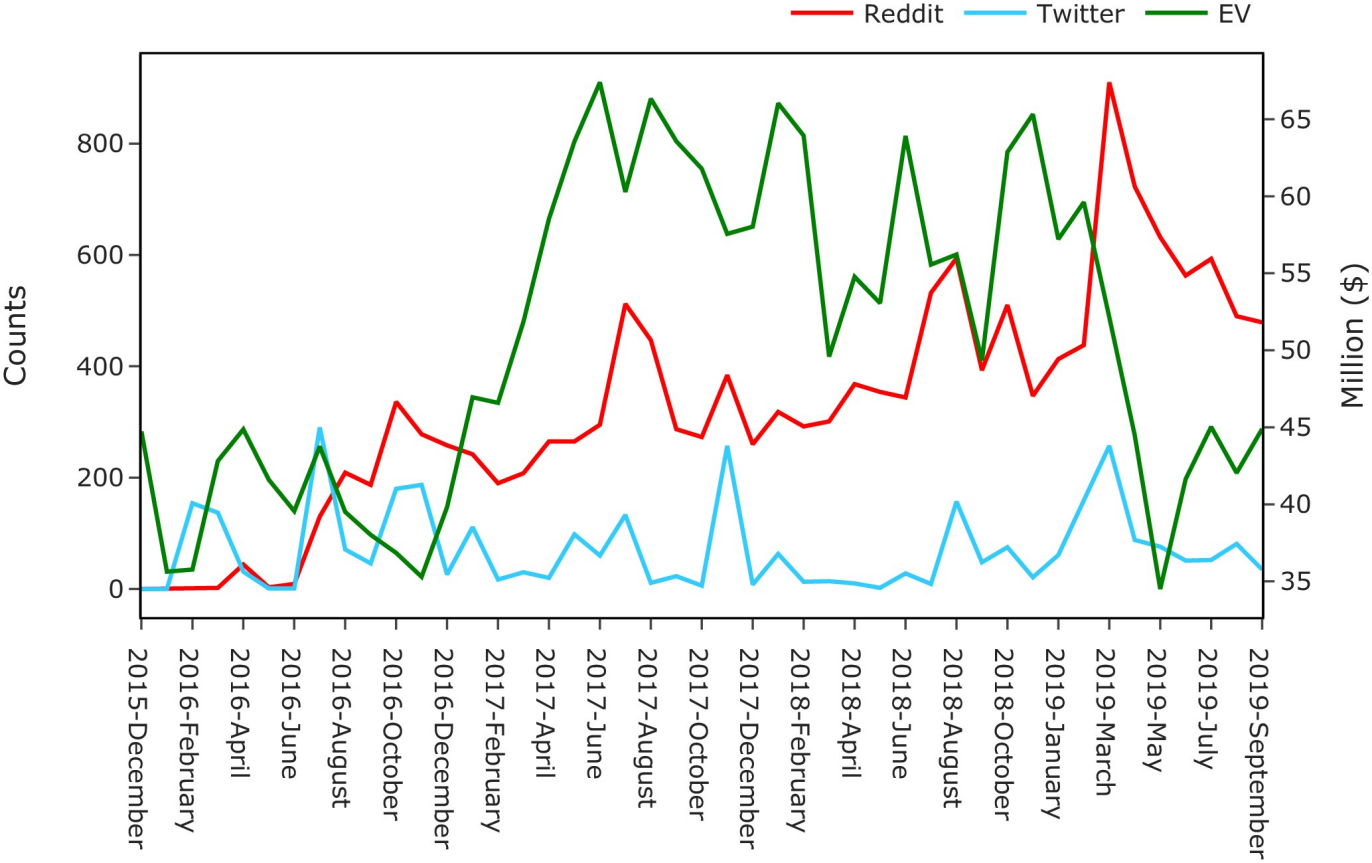
Tesla, Inc (NASDAQ: TSLA): Time-series trends of enterprise value (EV, green line, shown on right y-axis) vs online attention (Reddit, red line, and Twitter blue line, shown on the left y-axis).

Here, we conduct a case study indicative of the link between the share of attention received on online social media and offline financial performances of companies. We build the Enterprise Value (EV) time series of electric vehicle manufacturer Tesla, Inc (NASDAQ: TSLA) between December 2015 and September 2019 and we present it in Fig 7 together with the attention series in Reddit and Twitter. Using the methodology outlined in Materials and Methods (and further detailed in the S1 File), we show that both the series for Twitter and Reddit are Granger-causal for the EV (see Materials and methods). In other words, this result indicates that social media attention is a leading indicator for trends in growth in investors re-evaluation of Tesla’s value—i.e. the past online attention is predictive for the changes in enterprise value in the future. Furthermore, we can identify the ‘optimal’ lag between social attention and EV to being four months for Twitter and twelve months for Reddit.

This final result is significant, as it indicates a pathway of translating the increasing concentration of online attention to a handful of companies with online presence to an increasing dominance of these companies in the offline world given their boosted financial position. Of course, a wide array of factors determines the success of companies in the offline world, but we posit that the concentration of online attention might prove to be the one extra boost that can make the difference between growth and failure down the line.

## Conclusion

This study addresses an apparent paradox: the web is a source of continual innovation [44], and yet it appears increasingly dominated by a small number of dominant players [45]. This research tackles this paradox by using large-scale longitudinal data sets from social media to measure the distribution of attention across the whole online economy over more than a decade from 2006 until 2017. Here, we use outbound weblinks towards distinguishable web domains as a proxy for the market for online attention. As this data collection captures longitudinal trends relating to a universe of all potential websites and services, it serves as a valuable index of broader economic trends, dynamics, and patterns emerging online.

In this work, we provided evidence consistent with a link between increasing online attention on social media and the emergence (and growing) dominance of a small number of players. However, the question remains open concerning the real causes of dominance: is online attention part of them or just an early indicator? While it is impossible to infer causal relations from large observational data, such as those used here, our results are consistent with a putative link.

The development of the web has been steady, and it came in functional waves, each of which has been predicated by the emergence of foundation platform technologies— such as secure encryption, enabling e-commerce; ubiquitous broadband, enabling the emergence of streaming video and mobility, enabling the emergence of many new functions including car sharing. This research outlines that while new functions, services, and business models continuously emerge online, the web dynamics are such that in many mature categories of online services, one or a small number of competitors dominate. Yet, as new web technologies continue to be developed, this enables more unexplored functional niches to emerge and for the cycle to repeat [5]. Over time, this process leads to long-term declines in the overall competition, diversity, and decreasing survival rates for new entrants.

The world’s largest companies are now those that run global online platforms: Apple, Facebook, Google, and Amazon in the west and their counterparts Alibaba, Baidu, and Tencent in China. There is a growing public interest in the nature and extent of dominance on the web and web giants’ influence on economics, popular culture, and even politics. This paper extends understanding of the nature and scope of the web’s network effects on the evolution of businesses today. This work also opens the door to further research that uses digital footprints of organisations *en masse* as a basis for analysis of the behavioural economics and competitive dynamics of markets online. There is room here too for further work in simulation extending previous work done in synthetic market experimentation and prediction. [22, 46].

While Twitter and Reddit might not be representative for the web activity worldwide, they serve as a good representative sample of activity of most activity online in the English speaking web. Future work could compliment the analysis with equivalent datasets of online ecosystems in China (www.weibo.com, www.weechat.org), Russia (www.vk.com, www.yandex.com) and others.

As the web is an integral part of every industry, this research’s scope extends beyond technology firms [47–49]. Although the world’s largest technology giants are now also the largest companies globally, the technology sector itself still represents a small but growing subset of the overall economy. However, the reach and impact of digitisation, and online information and services has been shown to impact over 98% of the entire economy [50]. And while the data used in this study (from links in billions of online posts) reflects only the online activity, we posit that the patterns identified here represent the trends across the entire economy.

## Supporting information

S1 File (PDF)
