# Supplementary material for: Evolution of diversity and dominance of companies in online activity
Source: PLoS One. 2021 Apr 28;16(4):e0249993. doi: 10.1371/journal.pone.0249993 (PMC8081211; doi:10.1371/journal.pone.0249993)
Supplement: S1 File — (PDF) [file pone.0249993.s001.pdf]

S1 File

S1 Appendix: sampling social media data

Twitter sampling. HHI calculations in Economics typically involve looking at the market concentration of the top 50 companies in an industry or sector. With respect to the web as a whole, there are between ten and twenty major functions that have emerged including search, video, music, retail etc. Consequently sampling to include the concentration of the Top 1000 should correspond to covering leaders of the 10-20 most popular functions. Also Twitter is known to be a noisy data source. Because of the open API, many robots and re-tweeting tools are in operation which results in lots of links to sites and services that are non-mainstream. The large-scale global independent audience measurement service Quantcast[1] was used to determine the Top-1000 websites and HHI within this sample from twitter was used to measure the concentration of promotional robots who post large volumes of links to services that are not that well visited.

Representativeness of the Reddit dataset Here we report on the representativeness of our Reddit dataset. We used Amazon's Alexa, which ranks websites according to their traffic worldwide. The most visited websites are not the same as the most linked to. There is consistent overlap with the most popular sites, with Youtube, Snapchat and the BBC being among the most visited and linked to. However, there are other websites that are popular, but not that frequently linked to such as advertising networks, adult sites and non-English language websites. Conversely there are a variety of websites that attract large volumes of links, but which do not necessarily have many direct users. Examples include automated services that enable delayed or buffered posting of social media content, reposting of content from other social media and potentially promotional robots who post large volumes of links to services that are not that well visited.

We verify our samples' coverage by comparing them to the most popular sites on the web. S4 Fig shows that the majority of the most visited sites on the web are included in our sample with 95 percent of the Top 100 most visited domains in the world in 2016[2] are represented in our sample. Those that are missing comprise shortened URLs (that are later expanded) and selected advertising services and adult websites that are not widely shared among peers.

S2 Appendix: Enterprise Financial Performance Data

A study on the financial performance of companies is beyond the scope of this paper. Still, in the main text and Section S3 Appendix: detailed analysis of the link between social media attention and enterprise value, we examine the relationship between attention on social media and firm performance of one exemplar company (Tesla) in operating in a new and emerging category (electric vehicles). Links on social media are a significant measure of user attention, which can often be a precursor of revenue growth, a condition of financial performance. Previous research has illustrated that in new and emerging product categories, such as electric vehicles, competing brands' market share can be predicted by the percentage of links [14]. Tesla is now a pioneer in electric vehicles and is perceived as a technology leader in its own right. In 2020, Tesla overtook all other carmakers in terms of its enterprise value (as measured by market capitalisation) to become the world's most valuable car company[3]. As Tesla's current production volumes, revenue and earnings are significantly lower than other large global

[1] Quantcast global audience: `www.quantcast.com/`

[2] Source: Amazon's Alexa's Top 1 Million Most Visited Global Websites, 30th October 2016.

[3] `https://www.forbes.com/sites/sergeiklebnikov/2020/07/01/tesla-is-now-the-worlds-most-valuable-car-company-with-a-valuation-of-208-billion/?sh=6742e4d55334`

carmakers, this valuation is based largely on investors expectations of future earnings potential and growth of the company.

Preceding this rise in valuation, Telsa's brand has also attracted a more significant share of Wikipedia page visits since 2017 than its rivals (all top 10 carmakers[4], shown in Fig. S5 FigA). Currently, Tesla attracts more Google searches in the US than all other rivals except Toyota, having overtaken Ferrari in 2012, GM in 2013, and VW in 2016 (shown in Fig. S5 FigB). Our extended analysis (detailed in the following Section S3 Appendix: detailed analysis of the link between social media attention and enterprise value) shows that growth in the number of links in social media in both Reddit and Twitter to `Tesla.com` precedes an increase in the enterprise value of Tesla and that this link is Granger-causal. Together, these provide indirect evidence of the connection between online attention and offline market value of companies.

S3 Appendix: detailed analysis of the link between social media attention and enterprise value

In this section we provide the technical details on testing whether there is a granger-causal relation between online social media attention (Reddit and Twitter) and the enterprise value. Our analysis consists of two steps: first, testing the stationarity of the series and, second, performing the granger-causality test and determining the optimal lag value.

Step 1: Stationarity and co-integration tests To test the robustness of statistical forecasting using these data, we perform stationarity and co-integration on each of the data series shown in Fig. 7 (Reddit, Twitter, and Enterprise Value). We leverage an augmented Dickey-Fuller test (ADF) to test stationarity, in which the null hypothesis is that it exists a unit root in a time series (i.e., no stationarity) and the alternate hypothesis that the time series is stationary. S2 Table summarises the ADF statistic values and p-values of the three series related to Tesla. The results indicate that we cannot reject the null hypothesis for the Reddit and EV series (p-values $> 5\%$), and only the Twitter series is stationary. We transform the series by computing the difference between adjacent values. Consequently, each series is transformed into the series of 'difference from the previous month'. Applying the same ADF testing shows that the differences series are all stationary (see S2 Table). However, there are no long-term, predictable patterns for a stationary time series, and the co-integration test could help analyse the original non-stationary time series. Engle-Granger two-step method is called to test the stationarity of error term from the linear combination of those variables. The social media attention series (Reddit and Twitter) and the enterprise value series are not co-integrated based on the results in S2 Table. Based on the above two tests on Tesla, the stationary difference time series are used for the following analysis.

Step 2: Granger Causality and VAR Model. Granger causality test is used to examine whether social media data (Reddit & Twitter) can be used to forecast the changes in enterprise value. The null hypothesis is that accounting for lagged values of the social media series does not add explanatory power in predicting the enterprise value of Tesla. According to the results in S3 Table, we can reject the null hypothesis and conclude that the Reddit series is granger-causal for the enterprise value of Tesla for lags between 2 and 12 months. Similarly, the Twitter series has granger causality relation with Tesla EV for lags between 4 and 12 months. Based on the results, we can conclude that the fluctuations of social media datasets (such as link counts of Reddit and Twitter) explains some changes of enterprise value of Tesla during the period (2016

[4]Top 10 carmakes: `https://www.interbrand.com/best-global-brands/?filter-brand-sector=automotive`

Jan and 2019 Sep).

We explore the ‘optimal’ lag in the Reddit and Twitter series to best predict the EV series. The Vector Auto-Regression (VAR) model forecasts multiple time series variables using a regression model through their lagged vectors. We use VAR to determine the optimal lag for predicting the time series of Tesla by selecting the one with the lowest AIC criterion out of the valid options (for which the granger-causality test is significant). S4 Table summarises the AIC for up to 12 lags, and shows that a lag of 2 months for Reddit and 4 months for Twitter obtain the minimum AIC. It indicates that the online social media attention is predictive for the enterprise value for Tesla up to four months in advance (for Twitter) and up to one year in advance (for Reddit).

S4 Appendix: Goodness of fit for long-tail distributions

The total online attention of social media from different platforms (Reddit and Twitter) demonstrate different trends (see S2 Fig). The online volume of Reddit steadily increases after 2011 while there is a peak of attention at Twitter platform around 2016. It also explains the changes of the number of links for domains over time at Fig. 2.

We compare the goodness of fit of three distributions, power law, lognormal and exponential distribution, to find the best description of observed Reddit and Twitter data respectively. A Python package named ”powerlaw” is introduced to fit those distributions based on the estimated minimal distance between the theoretical distribution and the empirical data using the Kolmogorov-Smirnov test (see S3 Fig). The CCDF plots of power law and lognormal distribution are close to the empirical data while the plot of exponential distribution is far away from others.

Then it also provides the method to measure the log likelihood ratio between two different referenced distributions where is a ratio of probability that the observed data comes from the referenced distribution. Since we apply logarithmic scale on the ratio, positive ratios mean the probability of first theoretical distribution fit to data is higher than the probability of second distribution, otherwise the second distribution is preferred. S1 Table illustrates that most of the log likelihood ratios between power law versus lognormal or exponential distribution over all possible periods for Reddit and Twitter are positive. Even though the ratio of power law versus lognormal distrbution is negative in 2016 and 2017, the overall conclusion that the power law distribution is preferred to describe the observed social media data is consistent overall.

S1 Fig. Popularity in Twitter doesn't always correspond to broader population online. In this plot, we show the top domains by linked frequency within Twitter by month. Those shaded in dark green are Arabic-language sites, that perhaps are automated prayer reminder services and the light grey sites are automated re-tweeting services to help repost material from other social platforms such as Facebook. While popular in the number of links appearing on Twitter - these sites are not popular on the broader web. The right hand column with blue bars indicates the Quantcast Global ranking in of each of the domains in the last column for January 2017. Quantcast is an online audience measurement service that covers over 100 million web destinations and provides estimates of relative visitor traffic of all major global websites. The longer the blue bar, the higher the Quantcast rank, i.e. the lower the global popularity. Many of the webs most popular sites are present: Facebook (ranked 2nd); Youtube (4th) and Instagram (55th) however many of the high ranking sites by the number of links are not also popular on the broader web.

perc_54	X2016.07.15	perc_55	X2016.08.15	perc_56	X2016.09.15	perc_57	X2016.10.15	perc_58	X2016.11.15	perc_59	X2016.12.15	perc_60	X2017.01.15	perc_61	Rank2
25%	twitter.com	26%	twitter.com	27%	twitter.com	26%	twitter.com	26%	twitter.com	28%	twitter.com	27%	twitter.com	29%	6
6%	facebook.com	6%	facebook.com	6%	facebook.com	7%	facebook.com	6%	facebook.com	6%	facebook.com	6%	dua3a.org	6%	340906
5%	youtube.com	5%	youtube.com	5%	youtube.com	6%	youtube.com	6%	youtube.com	5%	youtube.com	5%	facebook.com	6%	2
4%	dua3a.org	5%	dua3a.org	5%	dua3a.org	4%	dua3a.org	4%	dua3a.org	4%	dua3a.org	5%	youtube.com	5%	4
3%	instagram.com	3%	instagram.com	3%	instagram.com	3%	instagram.com	3%	instagram.com	3%	instagram.com	3%	instagram.com	3%	55
2%	dlvr.it	2%	ift.tt	2%	ift.tt	2%	vine.co	2%	dlvr.it	2%	ift.tt	2%	dlvr.it	2%	465925
2%	vine.co	2%	dlvr.it	2%	dlvr.it	2%	ift.tt	2%	ift.tt	2%	dlvr.it	2%	ift.tt	2%	17766
2%	ift.tt	2%	vine.co	2%	vine.co	2%	dlvr.it	2%	d3waapp.org	2%	d3waapp.org	2%	d3waapp.org	2%	489767
1%	twimg.com	1%	d3waapp.org	1%	d3waapp.org	2%	d3waapp.org	2%	vine.co	2%	vine.co	1%	vine.co	1%	3120
1%	d3waapp.org	1%	7asnat.com	1%	7asnat.com	1%	7asnat.com	1%	7asnat.com	1%	7asnat.com	1%	curiouscat.me	1%	183576
1%	7asnat.com	1%	ln.is	1%	ln.is	1%	ln.is	1%	ln.is	1%	ln.is	1%	ln.is	1%	88480
1%	ln.is	1%	twimg.com	1%	bnent.jp	1%	bnent.jp	1%	bnent.jp	1%	viralsmesh.com	1%	ghared.com	1%	334225
1%	gph.to	1%	twcm.me	1%	twcm.me	1%	gigam.es	1%	gigam.es	0%	bnent.jp	1%	7asnat.com	1%	308642
1%	twcm.me	1%	buff.ly	1%	gigam.es	1%	curiouscat.me	0%	fllwrs.com	0%	curiouscat.me	1%	zad-muslim.com	1%	86759
1%	me2.do	1%	gigam.es	0%	buff.ly	1%	tistory.com	0%	zad-muslim.com	0%	twcm.me	1%	bnent.jp	1%	232026
0%	gigam.es	1%	bnent.jp	0%	curiouscat.me	0%	buff.ly	0%	buff.ly	0%	tistory.com	0%	tistory.com	0%	218351
0%	buff.ly	1%	zad-muslim.com	0%	zad-muslim.com	0%	zad-muslim.com	0%	ghared.com	0%	prt.nu	0%	prt.nu	0%	515891
0%	bnent.jp	0%	swarmapp.com	0%	swarmapp.com	0%	twcm.me	0%	tistory.com	0%	fllwrs.com	0%	ebay.com	0%	8
0%	swarmapp.com	0%	cas.st	0%	ghared.com	0%	ghared.com	0%	etsy.me	0%	zad-muslim.com	0%	buff.ly	0%	1006
0%	zad-muslim.com	0%	ghared.com	0%	tistory.com	0%	swarmapp.com	0%	twcm.me	0%	buff.ly	0%	totobet1.com	0%	450022
0%	ghared.com	0%	etsy.me	0%	cas.st	0%	fllwrs.com	0%	curiouscat.me	0%	ghared.com	0%	fllwrs.com	0%	52693
0%	etsy.me	0%	amazon.com	0%	amazon.com	0%	amazon.com	0%	twimg.com	0%	gigam.es	0%	twcm.me	0%	354712
0%	cas.st	0%	soundcloud.com	0%	nicovideo.jp	0%	etsy.me	0%	swarmapp.com	0%	etsy.me	0%	amazon.com	0%	3
0%	amazon.com	0%	tistory.com	0%	etsy.me	0%	cas.st	0%	amazon.com	0%	ebay.com	0%	gigam.es	0%	114271
0%	soundcloud.com	0%	fllwrs.com	0%	soundcloud.com	0%	soundcloud.com	0%	prt.nu	0%	swarmapp.com	0%	swarmapp.com	0%	43948
0%	fllwrs.com	0%	nicovideo.jp	0%	fllwrs.com	0%	nicovideo.jp	0%	ebay.com	0%	amazon.com	0%	soundcloud.com	0%	8898
0%	tistory.com	0%	curiouscat.me	0%	freebet.ga	0%	bett79.com	0%	soundcloud.com	0%	nicovideo.jp	0%	shindanmaker.com	0%	16371
0%	ask.fm	0%	giphy.com	0%	google.com	0%	twimg.com	0%	cas.st	0%	soundcloud.com	0%	nicovideo.jp	0%	6247
0%	nicovideo.jp	0%	tumblr.com	0%	tumblr.com	0%	betman7.com	0%	nicovideo.jp	0%	lol010.com	0%	periscope.tv	0%	18430
0%	tumblr.com	0%	ask.fm	0%	shindanmaker.com	0%	tumblr.com	0%	shindanmaker.com	0%	tumblr.com	0%	cas.st	0%	32108
0%	path.com	0%	shindanmaker.com	0%	ebay.com	0%	shindanmaker.com	0%	tumblr.com	0%	cas.st	0%	tumblr.com	0%	70623
0%	shindanmaker.com	0%	path.com	0%	ask.fm	0%	singapol.cf	0%	ameblo.jp	0%	totobet1.com	0%	bigo.sg	0%	444873
0%	periscope.tv	0%	imgur.com	0%	ameblo.jp	0%	ameblo.jp	0%	google.com	0%	ameblo.jp	0%	google.com	0%	1
0%	ebay.com	0%	ameblo.jp	0%	path.com	0%	imgur.com	0%	imgur.com	0%	periscope.tv	0%	ameblo.jp	0%	8495
0%	imgur.com	0%	wordpress.com	0%	twimg.com	0%	prt.nu	0%	wordpress.com	0%	bigo.sg	0%	imgur.com	0%	67
0%	ameblo.jp	0%	gph.to	0%	periscope.tv	0%	google.com	0%	periscope.tv	0%	shindanmaker.com	0%	wordpress.com	0%	33
0%	wordpress.com	0%	freebet.ga	0%	imgur.com	0%	ebay.com	0%	viralsmesh.com	0%	google.com	0%	boss-77.com	0%	238617

S2 Fig. Total online attention of Reddit and Twitter vary over time. (A) The monthly volume of links Reddit constantly increases from 2006 to 2019. **(B)** The monthly volume of links in Twitter (measured from 2011 to 2019) peaks in 2017 and decays ever since. The large variations are due to data crawling errors (i.e. periods in which our crawler does not record any data). The shaded area shows the 95% confidence level interval for predictions from a linear model.

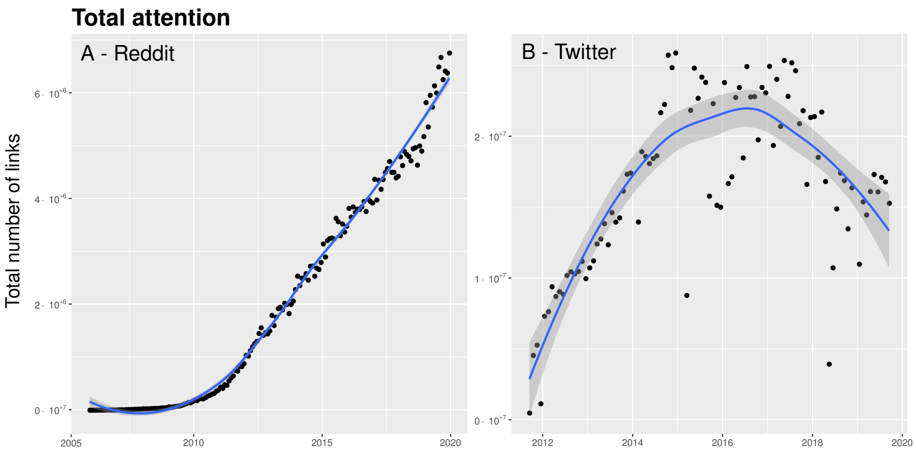

S3 Fig. Comparison of three distributions (i.e. power-law, lognormal and exponential distribution) fitted to Social Media (Reddit and Twitter) in 2016. The log-log plots of the empirical and fitted Complementary Cumulative Distribution Function (CCDF) of the number of links associated with domains in Reddit **(A)** and in Twitter **(B)**. Black solid lines represent the CCDF of observed data while other dashed lines represent the power-law (red), lognormal (green) and exponential (blue) fitted distribution.

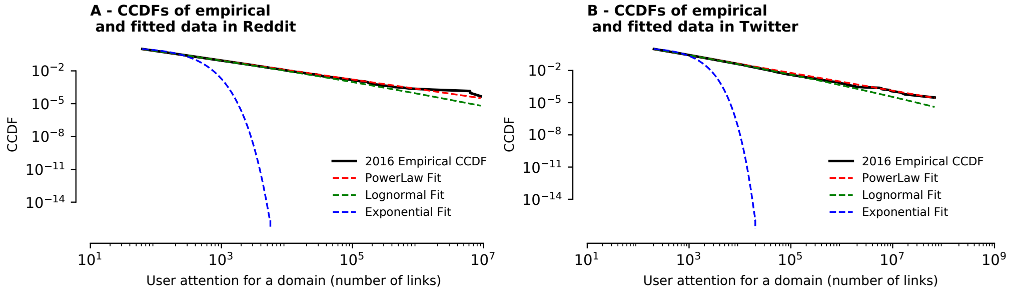

S4 Fig. The overwhelming majority of the top most visited web sites in the world (according to the Alexa ranking) are included in our data collection. *(left)* 95 of the top 100 are present in our Reddit collection; those missing are concatenations or short-form websites such as Bit.ly, as well as a small number of Advertising network sites and Adult sites that are popular but not shared. *(right)* The top Alexa websites account for a greater proportion of all Reddit links over time. The Top 1000 ranked websites accounted for less than 60% of all posts in 2010 and almost 70% in 2016.

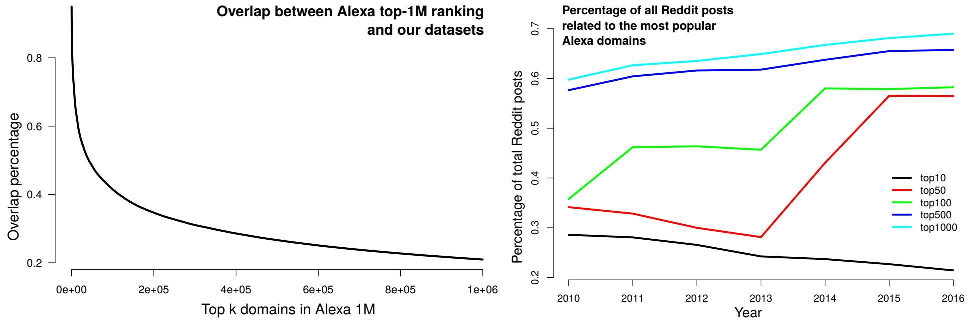

S5 Fig. Online attention for major car manufacturers. Tesla overtakes its competition. **(A)** Wikipedia Page View per month. We collect the monthly page views of the carmakers' pages, from 2017 until 2021. The y-axis shows the page views, and visibly Tesla's page is viewed consistently more than its competitors. **(B)** Google searches in the US. We use Google search trends to plot Google searches in the US for car manufacturers from 2004 until 2021. The y-axis shows the search volume in each month, in percentage, relative to the highest value recorded for any of the manufacturers (here, Toyota in Sept 2019). Visibly, Tesla has consistently increased since 2013 and had overtaken all competitors except Toyota.

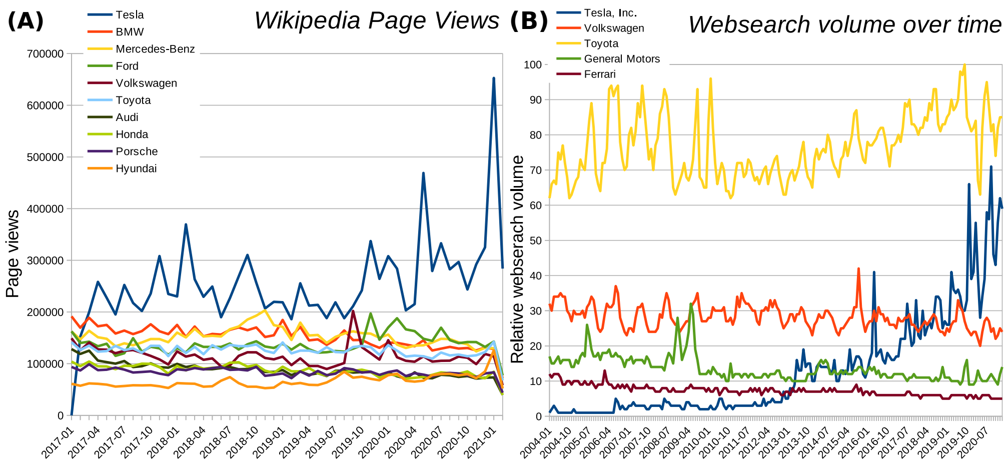

(A)
Wikipedia Page Views
Tesla
BMW
Mercedes-Benz
Ford
Volkswagen
Toyota
Audi
Honda
Porsche
Hyundai
Page views
(B)
Websearch volume over time
Tesla, Inc.
Volkswagen
Toyota
General Motors
Ferrari
Relative webserach volume

S1 Table Log likelihood ratios of power-law vs. lognormal and exponential distribution, for Reddit and Twitter. Positive ratios indicated that power-law is prefered to describe Social Media data. Conversely, negative values indicate that lognormal/exponential distribution is preferred.

Year	Reddit		Twitter	
	Power law vs. Lognormal	Power law vs. Exponential	Power law vs. Lognormal	Power law vs. Exponential
2006	1.422	1.799	-	-
2007	2.767	2.457	-	-
2008	1.467	2.449	-	-
2009	2.933	2.690	-	-
2010	1.373	2.632	-	-
2011	2.007	2.734	3.637	4.288
2012	3.261	3.067	1.127	3.446
2013	2.655	3.026	2.395	3.918
2014	1.673	2.781	0.203	5.767
2015	0.835	2.744	0.395	3.862
2016	1.552	2.874	-2.554	2.930
2017	1.765	2.771	-6.382	2.382
2018	3.892	2.789	1.048	1.641
2019	2.643	2.341	1.618	1.476

S2 Table Summary of the stationarity and the co-integration tests, for linking social media attention and enterprise value for Tesla: ADF statistics and p-values results show that the Reddit and EV series are not stationary. However, the first difference of the series (i.e the difference between two adjacent values) is stationary for all variables (Reddit, Twitter and EV). The co-integration test indicates no co-integration between Reddit and EV, and Twitter and EV respectively.

Tesla	Time Series	ADF Statistics	P-value
Stationarity Test	Link counts in Reddit	-0.19	46.80%
	First difference Reddit	-3.20	2.01%*
	Link counts in Twitter	-6.40	0.00%*
	First difference Reddit	-4.68	0.00%*
	Enterprise Value	-1.99	28.68%
	First difference in EV	-7.48	0.00%*
Co-integration Test	Error term of Reddit & EV	-2.09	24.91%
	Error term of Twitter & EV	-2.20	20.49%

S3 Table Summary of the results of Granger-causality test between social media attention and the enterprise value. The lag column indicate the maximum lag of time included in the Granger-causality test. The Reddit and Twitter columns show the corresponding p-value of Granger test of the respective social media platform and the enterprise value series. Values prefixed by asterisk (*) indicate statistical significance with p-value < 5%. Results indicate that the enterprise value is granger-caused by both Reddit (after a lag of 2) and Twitter (after a lag of 4).

Lag	Reddit	Twitter
1 month	24.64%	46.07%
2 months	1.32%*	63.71%
3 months	2.41%*	54.62%
4 months	0.96%*	0.30%*
5 months	0.40%*	0.52%*
6 months	0.03%*	0.02%*
7 months	0.04%*	0.04%*
8 months	0.06%*	0.09%*
9 months	0.00%*	0.11%*
10 months	0.00%*	0.08%*
11 months	0.01%*	0.00%*
12 months	0.00%*	0.00%*

S4 Table The value of the AIC criterion of VAR model between Twitter and Enterprise Value of Tesla, for lags values between 0 and 12 months. We chose as the 'best lag' the lag that minimises the AIC (indicated by *) from the set of valid values (for which the granger-causality test is significant, shown in **bold face**).

Lag	Reddit	Twitter
0	*13.15*	*12.97*
1 month	*13.21*	*12.90*
2 months	**13.25**	*12.97*
3 months	**13.36**	*12.84*
4 months	**13.31**	**12.70***
5 months	**13.31**	**12.91**
6 months	**13.29**	**12.92**
7 months	**13.48**	**12.94**
8 months	**13.69**	**13.14**
9 months	**13.83**	**13.40**
10 months	**13.75**	**13.49**
11 months	**13.74**	**13.41**
12 months	**13.01***	**13.40**

S5 Table Rivalfox data. The most linked global online service (shown in **bold face**) in twelve of the functional categories defined by Crunchbase, and their three main direct rivals identified by Rivalfox.

Category	Domain	Company	Year started
Video	vimeo.com	Vimeo	2004
Video	www.truveo.com	Truveo	2004
Video	www.dailymotion.com	Dailymotion	2005
Video	www.youtube.com	**Youtube**	2005
Filesharing	drive.google.com	Google Drive	2012
Filesharing	home.elephantdrive.com	Elephant Drive	2005
Filesharing	www.sugarsync.com	Sugarsync	2004
Filesharing	www.dropbox.com	**Dropbox**	2007
Accommodation	www.homeaway.com	HomeAway	2005
Accommodation	www.wimdu.com	Wimdu	2011
Accommodation	www.9flats.com	9flats	2012
Accommodation	www.airbnb.com	**AirBnb**	2008
Music streaming	hello.simfy.de	simfy	2006
Music streaming	www.rdio.com	Rdio	2008
Music streaming	www.deezer.com	Deezer	2006
Music streaming	www.spotify.com	**Spotify**	2008
Ride sharing	www.lyft.com	Lyft	2007
Ride sharing	www.hailoapp.com	Hailo	2010
Ride sharing	www.side.cr	Sidecar	2010
Ride sharing	www.uber.com	**Uber**	2009
Search	www.bing.com	Bing	2009
Search	www.duckduckgo.com	DuckDuckGo	2008
Search	www.yahoo.com	Yahoo!	1994
Search	www.google.com	**Google**	1998
Social Network	twitter.com	Twitter	2006
Social Network	plus.google.com	Google Plus	1998
Social Network	tumblr.com	Tumblr	2007
Social Network	facebook.com	**Facebook**	2004
General Retail	amazon.com	**Amazon**	1994
General Retail	walmart.com	Walmart	1962
General Retail	ebay.com	eBay	1995
General Retail	bestbuy.com	Best Buy	1966
Movies	Veed.me	Veed	2013
Movies	hulu.com	Hulu	2007
Movies	netflix.com	**Netflix**	1997
Movies	amazon.com/video	Amazon video	2006
Ephemeral messaging	wickr.com	Wickr	2011
Ephemeral messaging	clipchat.com	Clipchat	2013
Ephemeral messaging	sling.me	Slingshot	2014
Ephemeral messaging	snapchat.com	**Snapchat**	2011
Action cameras	contour.com	Contour	2004
Action cameras	eyesee360.com	EyeSee360	1998
Action cameras	vievu.com	Vievu	2007
Action cameras	gopro.com	**GoPro**	2003
Dating app	chatimity.com	Chatimity	2011
Dating Apps	hinge.co	Hinge	2011
Dating Apps	wyldfireapp.com	Wyldfire	2013
Dating Apps	gotinder.com	**Tinder**	2012
